# Characterization of the complete mitochondrial genome of *Parabreviscolexniepini* Xi et al., 2018 (Cestoda, Caryophyllidea)

**DOI:** 10.3897/zookeys.783.24674

**Published:** 2018-09-05

**Authors:** Bing-Wen Xi, Dong Zhang, Wen-Xiang Li, Bao-Juan Yang, Jun Xie

**Affiliations:** 1 Freshwater Fisheries Research Center, Chinese Academy of Fishery Sciences, 214081 Wuxi, China Freshwater Fisheries Research Center, Chinese Academy of Fishery Sciences Wuxi China; 2 Institute of Hydrobiology, Chinese Academy of Sciences, Wuhan 430072, China Institute of Hydrobiology, Chinese Academy of Sciences Wuhan China

**Keywords:** unsegmented cestode, eucestode, tapeworm, mitogenome, schizothoracine fish

## Abstract

*Parabreviscolexniepini* is a recently described caryophyllidean monozoic tapeworm from schizothoracine fish on the Tibetan Plateau. In the present study, the complete mitochondrial genome of *P.niepini* is determined for the first time. The mitogenome is 15,034 bp in length with an A+T content of 59.6%, and consists of 12 protein-encoding genes, 22 tRNA genes, two rRNA genes, and two non-coding regions. The secondary structure of tRNAs exhibit the conventional cloverleaf structure, except for *trnS1*^(AGN)^ and *trnR*, which lack DHU arms. The anti-codon of *trnS1*^(AGN)^ in the mitogenome of *P.niepini* is TCT. The two major non-coding regions, 567 bp and 1428 bp in size, are located between *trnL2* and *cox2*, *trnG* and *cox3*, respectively. The gene order of *P.niepini* shows a consistent pattern with other caryophyllideans. Phylogenetic analysis based on mitogenomic data indicates that *P.niepini* has a close evolutionary relationship with tapeworms *Breviscolexorientalis* and *Atractolytocestushuronensis*.

## Introduction

The Caryophyllidea is an ancient group of tapeworms, consisting of four families, 42 genera, and approximately 190 species parasitic in cypriniform and siluriform fishes in most zoogeographical regions (Scholz and Oros 2017). Some caryophyllideans, especially those in cyprinids (e.g. *Khawiasinensis* Hsü, 1935), cause severe fish diseases. The simplification and limited number of morphological characters cause species identification and taxonomic classification is problematic. Recent research found that the present classification of caryophyllideans could not reveal the natural phylogenetic relationships (Brabec et al. 2012; Xi et al. 2018). Further studies were desired to re-construct the taxonomic system. Maternal inheritance and rapid evolution have proven to be key factors in phylogenetic studies in tapeworms, making mitochondrial DNA a powerful marker for species identification (e.g. Brabec et al. 2012; Li et al. 2017).

*Parabreviscolex* Xi, Oros, Chen & Xie, 2018 is a recently erected genus in the family Capingentidae Hunter, 1930 (Cestoda: Caryophyllidea), with the type species *Parabreviscolexniepini* Xi, Oros, Chen & Xie, 2018 from schizothoracine fish on the Tibetan Plateau (Xi et al. 2018). The historical uplift of the Tibetan Plateau has caused significant differentiation of the Tibetan biotas, resulting in many endemic species. The evolution and adaptation processes of those species have attracted much attention. In this study, the complete mitogenome of *P.niepini* was sequenced, which may provide useful information for better understanding the evolution and taxonomy within caryophyllideans.

## Materials and methods

### Specimen collection and DNA extraction

*Parabreviscolexniepini* were collected from the schizothoracine fish *Schizopygopsisyounghusbandi* Regan, 1905 in the Yarlung Tsangpo River at Linzhi (29°39'N, 94°21'E), Tibet, China, and the specimens were fixed in 100% ethanol and stored at Freshwater Fisheries Research Center, Chinese Academy of Fishery Sciences. Total genomic DNA was extracted using a TIANamp Micro DNA Kit (Tiangen Biotech, Beijing, China), according to the manufacturer’s instructions. DNA was stored at -20 °C for further molecular analyses.

### PCR and DNA sequencing

The whole mitogenome was amplified with primers designed based on closely related tapeworms (Suppl. material 1). PCR reactions were performed in a 20 µL reaction mixture, containing 7.4 µL dd H_2_O, 10 µL 2×PCR buffer (Mg^2+^, dNTP plus, Takara, China), 0.6 µL of each primer (10 µM), 0.4 µL ExTaq polymerase (Takara, China), and 1 µL DNA template (200 ng/µL). Amplification was conducted as follows: initial denaturation at 95 °C for 2 min, followed by 40 cycles at 95 °C for 10 sec, 46 °C – 53 °C for 30 sec (annealing temperature depending on the primers used, see Suppl. material 1), and 68 °C for 90 sec, and final extension at 68 °C for 10 min. PCR products were sequenced bidirectionally at Sangon Biotech (Shanghai, China) using the primer walking strategy.

### Sequence annotation and analyses

The amplified fragments were quality-proofed, and BLASTN (Altschul et al. 1990) to confirm the fragments were the actual target sequence. The complete mitochondrial genomic sequence of *P.niepini* was assembled manually in a stepwise manner using the DNAstar v7.1 program (Burland 2000). To determine the gene boundaries, it was aligned against the reference mitogenomic sequences of *Atractolytocestushuronensis* Anthony, 1958 (KY486754) using the program MAFFT 7.149 (Katoh and Standley 2013) integrated with Geneious (Kearse et al. 2012). The mitogenome was annotated and characterized mainly following previous descriptions (Zhang et al. 2017a, b; Zou et al. 2017; Li et al. 2018). Protein-coding genes (PCGs) were found by searching for ORFs (employing genetic code 9, an echinoderm mitochondrial genome) and checking the nucleotide alignments against the reference genome in Geneious. All tRNAs were identified, and confirmed with ARWEN (Laslett and Canback 2008) and MITOS (Bernt et al. 2013) web servers. Similarly, *rrnL* and *rrnS* were initially found using MITOS and their boundaries were determined by the alignments with the reference genome in Geneious. The NCBI submission file and tables with statistics for mitogenomes were generated using a GUI-based program, MitoTool (Zhang 2016b). Tandem Repeats Finder (Benson 1999) was employed to find tandem repeats in the non-coding regions. A nucleotide composition table was then used to make the broken line graph of A+T content in ggplot2 (Hadley 2009). Codon usage and relative synonymous codon usage (RSCU) for twelve protein-encoding genes (PCGs) of *P.niepini* was computed and sorted using MitoTool, and finally imported to ggplot2 to draw the RSCU figure. Ggplot2 was used to draw scatter diagrams for the principal component analysis (PCGs) and nucleotide skews. Input files for the PCGs of codon usage pattern, as well as analyses of amino acid usage pattern and nucleotide skews, were generated by MitoTool. PASW 18.0 (Allen and Bennett 2010) was used to conduct a principal component analysis and generate data for the scatter diagram.

### Phylogeny and gene order

Phylogenetic analyses were undertaken using nucleotide sequences of all 36 genes of the newly sequenced mitogenome of *P.niepini* and 36 selected cestodes mitogenomes available in the GenBank (Suppl. material 2). The mitogenomic sequences of *Khawiasinensis* (NC_034800/KR676560) and *Caryophyllaeusbrachycollis* Janiszewska, 1953 (NC_035430/KT028770) from the common carp, sequenced and deposited in GenBank by the same researchers (Feng et al. 2017), were reassigned herein as *Khawia* sp. 1 and *Khawia* sp. 2, respectively, because the species identifications were questionable. *Caryophyllaeusbrachycollis* mainly infests the cyprinid *Barbus* and *Abramis* in European countries, while its occurrence in China is rare (Barčák et al. 2014). We considered that the researchers have misidentified the two common tapeworms *Khawiasinensis* and *Khawiajaponensis* (Yamaguiti, 1934) from the common carp.

Two trematode species, *Dicrocoeliumdendriticum* (Rudolphi, 1819) (NC_025280) and *Dicrocoeliumchinensis* Tang & Tang, 1978 (NC_025279), were used as outgroups. The nucleotide sequences for all 12 PCGs, two rRNAs and 22 tRNAs were extracted from GenBank files. The PCGs were translated into amino acid sequences (employing genetic code 9) using MitoTool, and aligned in batches with MAFFT integrated into another GUI-based program BioSuite (Zhang 2016a) using codon-alignment mode. RNAs were aligned with structural alignment mode using the Q-INS-i algorithm incorporated into MAFFT-with-extensions software. BioSuite was then used to concatenate these alignments and remove ambiguously aligned fragments from the concatenated alignments by another plug-in program, Gblocks 0.91b (Talavera and Castresana 2007). Phylogenetic analyses were conducted using maximum likelihood (ML) and Bayesian inference (BI) methods. Selection of the most appropriate evolutionary model for the dataset was carried out using ModelFinder (Kalyaanamoorthy et al. 2017). Based on the Bayesian information criterion, GTR+I+G was chosen as the optimal model for both analyses. ML analysis was performed in RaxML GUI (Silvestro and Michalak 2011) using a ML + rapid bootstrap (BP) algorithm with 1000 replicates. BI analysis was performed in MrBayes 3.2.6 (Ronquist et al. 2012) with default settings, and 6×10^6^ metropolis-coupled MCMC generations.

### Selection analyses

To determine lineage-specific positively selected sites in individual mitochondrial PCGs, a branch-site model incorporated by CodeML within PAML package (Yang 2007) was used. The resultant ML and/or BI tree (unrooted tree with outgroups removed) was employed for the analysis. The alternative model, MA fixes ω at 1 for each branch except for the specified branch leading to *P.niepini* (foreground branch), wherein ω is presumed to be greater than 1. The first null model MAnull fixes ω at 1 for every branch in the tree, whereas the second null model M1a fixes ω at 1 for every branch except for the foreground branch, where ω is assumed to be in the range 0 to 1. The null model and alternative model were compared via a likelihood ratio test (LRT), and positive selection was confirmed when P<0.05. Comparing MA to MAnull can estimate positive selection, while comparing MA to M1a can identify instances of relaxation of selective constraints as well as positive selection (Láruson 2017). The posterior probabilities value (≥ 95%) of Bayes Empirical Bayes (BEB) method was used to identify for positively selected sites (Yang 2005).

## Results

### Genome organization and base composition

**Figure 1.**
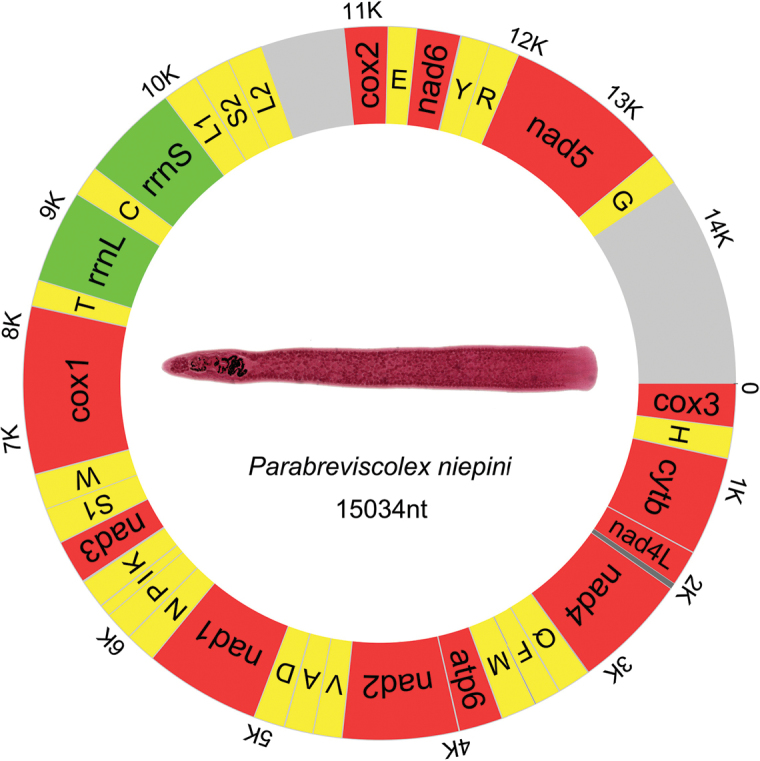
Circular representation of the mitochondrial genome of *Parabreviscolexniepini*. Different colors were used to indicated protein-coding genes (12) (red), tRNAs (22) (yellow), rRNAs (2) (green), and non-coding regions (grey). Tapeworm was stained with iron acid carmine.

**Figure 2.**
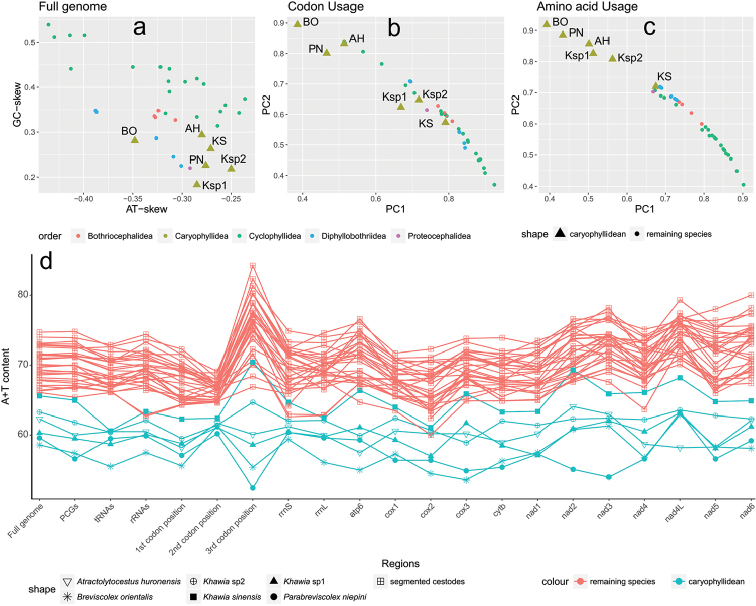
**a** The comparison of nucleotide skewness of the full genomes for the mitogenome of *Parabreviscolexniepini* and other cestodes **b, c** Principal component (PC) analysis of the codon usage and amino acid usage in the PCGs of *P.niepini* and other cestodes. The first PC (PC1) and the second PC (PC2) of the codon usage and amino acid usage accounted for 96.7% and 98.08% of the variability, respectively. **d** G+T content of complete genomes and their individual elements. The six caryophyllideans are represented by triangles in a-c. Abbreviations: AH: *Atractolytocestushuronensis*; BO: *Breviscolexorientalis*; Ksp2: *Khawia* sp. 2; KSK: *Khawiasinensis*; Ksp1: *Khawia* sp. 1; PN: *Parabreviscolexniepini*.

The closed-circular mitochondrial genome of *Parabreviscolexniepini* is 15,034 bp in size (GenBank accession number: MG674140). The mitogenome is composed of 12 protein-encoding genes (PCGs), 22 tRNA genes, two rRNA genes, two non-coding regions, and it lacks the *atp8* gene (NCR) (Fig. 1). As is common in flatworms, all genes are transcribed from the same strand (Le et al. 2002). Eight overlapping regions and 16 intergenic regions were found in the genome (Table 1). In accordance with other caryophyllidean species, the A+T content of the whole genome (59.6%) and its elements are lower than in the segmented cestodes (Fig. 2d). The mitogenome of *P.niepini* exhibits G-skew and T-skew, which is also the case in other cestodes (Fig. 2a). However, the unsegmented cestodes appear to exhibit less mutation bias than segmented cestodes (lower GC-skew and higher AT-skew values, Fig. 2a).

**Table 1.** Annotated mitochondrial genome of *Parabreviscolexniepini*.

Gene	Position		Size	Intergenic nucleotides	Codon		Anti-codon
	From	To			Start	Stop	
cox3	1	643	643		ATG	T	
trnH	644	706	63				GTG
cytb	710	1798	1089	3	ATG	TAG	
nad4L	1802	2062	261	3	ATG	TAG	
nad4	2023	3255	1233	-40	ATG	TAG	
trnQ	3252	3309	58	-4			TTG
trnF	3314	3378	65	4			GAA
trnM	3374	3436	63	-5			CAT
atp6	3440	3955	516	3	ATG	TAG	
nad2	3960	4832	873	4	ATG	TAG	
trnV	4835	4896	62	2			TAC
trnA	4895	4955	61	-2			TGC
trnD	4960	5021	62	4			GTC
nad1	5022	5915	894		GTG	TAG	
trnN	5915	5980	66	-1			GTT
trnP	5984	6045	62	3			TGG
trnI	6045	6109	65	-1			GAT
trnK	6114	6175	62	4			CTT
nad3	6185	6523	339	9	GTG	TAG	
trnS1	6522	6580	59	-2			TCT
trnW	6581	6646	66				TCA
cox1	6650	8210	1561	3	ATG	T	
trnT	8208	8273	66	-3			TGT
rrnL	8274	9226	953				
trnC	9227	9286	60				GCA
rrnS	9287	9993	707				
trnL1	9994	10059	66				TAG
trnS2	10063	10125	63	3			TGA
trnL2	10127	10190	64	1			TAA
cox2	10758	11330	573	567	ATG	TAG	
trnE	11331	11391	61				TTC
nad6	11392	11850	459		GTG	TAG	
trnY	11859	11923	65	8			GTA
trnR	11925	11982	58	1			TCG
nad5	11983	13542	1560		GTG	TAA	
trnG	13543	13606	64				TCC

### Protein-coding genes and codon usage

Coalesced PCGs were 9999 bp in size, the lowest A+T content (56.6%) in all selected eucestodes (Suppl. material 2), which is also reflected in individual PCGs from 54% (*nad3*) to 63.2% (*nad4L*) (Suppl. material 3). ATG is the most commonly used initial codon for eight PCGs; exceptions are *nad1*, *nad3*, *nad6*, and *nad5*, which use GTG. Among the terminal codons, nine out of 12 are TAG, while *nad5* uses TAA, *cox3*, and *cox1* uses abbreviated stop codons (T--) (Table 1).

Codon usage, RSCU, and codon family proportion (corresponding to the amino acid usage) of *P.niepini* was investigated (Suppl. material S5). The four most abundant codon families (Phe, Val, Leu2, and Gly) encompass 38.41% of all codon families. Among these codon families, G+T-rich codons are favored over synonymous codons with lower G+T content in *P.niepini* (Suppl. material S5). This G+T preference corresponds well with the relatively high G+T content (Suppl. material S3) as well as G and T preference in the skewness analysis for PCGs (Suppl. material S2). Additionally, the principal component analyses (PCGs) suggested that the overall amino acid usage patterns of the unsegmented cestodes (except for *Khawiasinensis*KY486753) were apparently different from segmented cestodes (Fig. 2c). Noteworthy, in contrast to segmented cestodes, which have notably heightened A+T content at the 3^rd^ codon position, these unsegmented cestodes (except *Khawiasinensis*KY486753) exhibit lower and/or similar A+T content to other elements of the mitogenome (Fig. 2d).

### Transfer and ribosomal RNA genes

The two rRNAs, *rrnL*, and *rrnS* are 953 and 707 bp in size, with 59.6% and 60.4% A+T content, respectively (Suppl. material S3). All 22 commonly found tRNAs are present in the mitochondrial genome of *P.niepini*, ranging from 58 bp (*trnQ* and *trnR*) to 66 bp in size (*trnN*, *trnW*, *trnT* and *trnL1*), and adding up to 1381 bp in total coalesced length (Table 1 and Suppl. material S2). All of the secondary structures (predicted by MITOS and ARWEN) exhibit the conventional cloverleaf structure, except for *trnS1*^(AGN)^ and *trnR*, which lack DHU arms. The unorthodox *trnS1*^(AGN)^ and *trnR* were also found in the Caryophyllidea (Li et al. 2017) and the Anoplocephalidae (Guo 2016). Additionally, the anti-codon of *trnS1*^(AGN)^ in the mitogenome of *P.niepini* is TCT, in contrast to other eucestodes, which use GCT, except for *Khawiasinensis* (KY486753) (Suppl. material S4).

### Non-coding regions

**Figure 3.**
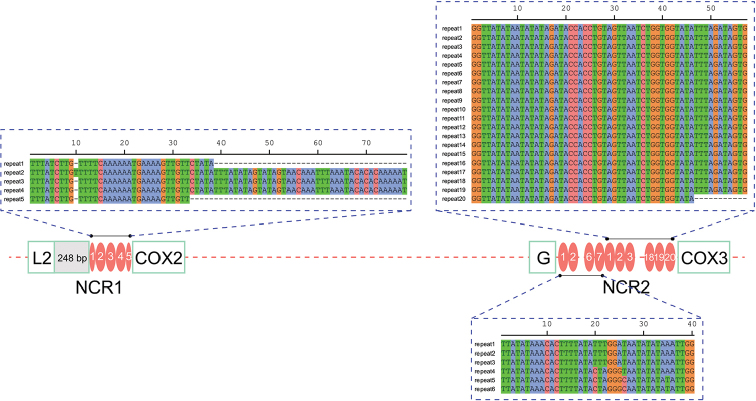
Tandem repeats in two main non-coding regions of *Parabreviscolexniepini*.

The two major non-coding regions (NCR), 567 bp (NCR1) and 1428 bp (NCR2) in size, are located between *trnL2* and *cox2* and between *trnG* and *cox3*, respectively. The positions of the two NCR are consistently reported in other unsegmented tapeworms (see fig. s3 of Li et al. 2017). They have apparently higher A+T content (76.5% for NCR1 and 72.6% for NCR2) than other parts of the genome (Suppl. material S3). The NCR1 contain five tandem repeats (TRs), with two truncated TRs (repeat unit 1 and 5) and one T insertion in repeat unit 2 (Fig. 3). Two highly repetitive regions (HRR) were found in NCR2. HRR1 possess seven TRs, identical in size (40 bp). Repeat units 1–3 are identical in nucleotide composition. In comparison to the repeat units 1–3, repeat unit 4 differed in three nucleotides, while unit 5 and 6 differed in five nucleotides (Fig. 3). HRR2 possess 20 TRs, repeat units 1–19 are identical in size (57 bp) and nucleotide composition, whereas unit 20 is 46 bp long (Fig. 3).

### Phylogeny and gene order

**Figure 4.**
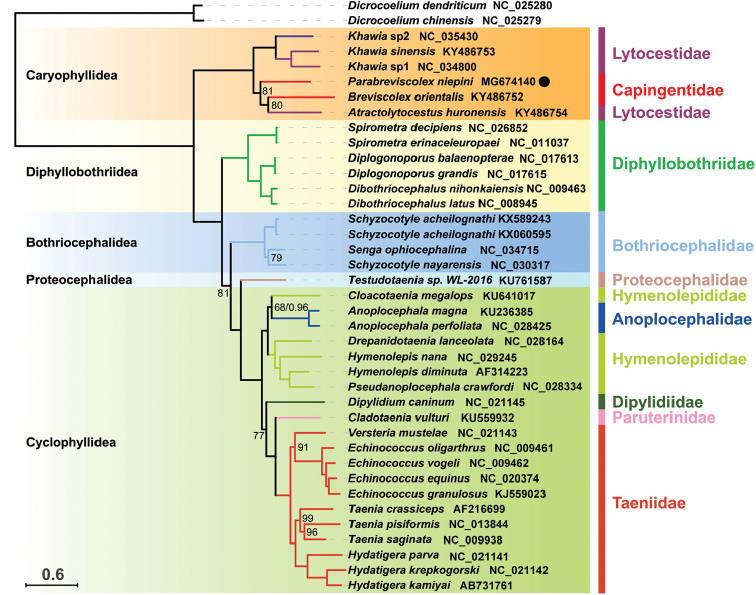
Phylogenetic tree of five cestode orders inferred from maximum likelihood analysis with concatenated nucleotide sequence of all 36 genes (12 PCGs, 2 rRNAs, and 22 tRNAs). Bootstrap (BS)/bayesian posterior probability (BPP) support values are shown above the nodes, only BS < 100 and BPP < 1 are displayed. Scale bar represents the estimated number of substitutions per site.

The phylogenetic topology constructed using BI and ML methods show concordant branches and high statistical support. All bootstrap support values (BS) are higher than 68 and Bayesian posterior probabilities (BPP) are higher than 0.96. *Parabreviscolexniepini* exhibits the closest phylogenetic relationship with *Breviscolexorientalis* Kulakovskaya, 1962 and *Atractolytocestushuronensis* with robust support (Fig. 4). Moreover, the similarity of the codon usage pattern (Fig. 2b) lends further support to the phylogenetic affinity of *P.niepini*, *B.orientalis* and *A.huronensis*. The mitochondrial gene arrangement of *P.niepini* (Fig. 1) shows a consistent pattern in the Caryophyllidea, and obviously differs from the segment tapeworms (fig. S3 of Li et al. 2017).

### Branch-site analysis

The branch-site model tests based on the criteria of posterior probabilities ≥ 95% in the BEB analyses and in the likelihood ratio test (LRT) (*P<*0.05), found the amino acid positions V(6) and H(49) of *P.niepinicytb* (Suppl. material S6, Table 2) were under positive selection. Moreover, several sites in *nad4*, *nad5*, and *cox3* were also identified to exhibit relaxed selective pressure (Table 2).

**Table 2.** Summary of branch-site model analyses for genes *cytb*, *nad4*, *nad5*, *nad2*, *cox3* and *cox1* of *Parabreviscolexniepini*.

Gene	Null model	Parameter estimated					P value	Positively selected sites (BEB analysis)
		Site class	0	1	2a	2b		
*cytb*	Model A null	proportion	0.86659	0.0458	0.08322	0.0044	p < 0.05^*^	6V 0.970/49H 0.975
		background w	0.02999	1	0.02999	1		
		foreground w	0.02999	1	13.90314	13.90314		
*nad4*	M1a	proportion	0.77596	0.18941	0.02783	0.00679	p < 0.05^*^	146S 0.974
		background w	0.0505	1	0.0505	1		
		foreground w	0.0505	1	11.95711	11.95711		
*nad5*	M1a	proportion	0.66652	0.24542	0.06436	0.0237	p < 0.01^**^	117A 0.961/212T 0.966/
		background w	0.0584	1	0.0584	1		303M 0.991
		foreground w	0.0584	1	2.01044	2.01044		
*nad2*	M1a	proportion	0.80968	0.09257	0.08772	0.01003	p < 0.01^**^	
		background w	0.03581	1	0.03581	1		
		foreground w	0.03581	1	97.38901	97.38901		
*cox3*	M1a	proportion	0.76066	0.06085	0.16527	0.01322	p < 0.01^**^	32A 0.954/65S0.989/
		background w	0.03801	1	0.03801	1		105Y 0.996/184T 0.957
		foreground w	0.03801	1	2.15924	2.15924		
*cox1*	M1a	proportion	0.93133	0.04455	0.02301	0.0011	p < 0.01^**^	
		background w	0.01974	1	0.01974	1		
		foreground w	0.01974	1	2.28474	2.28474		
*cytb*	M1a	proportion	0.86658	0.0458	0.08322	0.0044	p < 0.01^**^	6V 0.970/49H 0.975
		background w	0.02999	1	0.02999	1		
		foreground w	0.02999	1	13.90396	13.90396		

## Discussion

The different evolution rate of individual genes render phylogenetic analysis of cestode complicated or unreliable for some taxa; however, the complete mtDNA data was considered to provide the best interrelationship estimate (Waeschenbach et al. 2012). So far, the amount of mitogenome data available was limited. In this study, we sequenced and characterized the sixth mitogenome of caryophyllidean. The phylogenetic analysis constructed here placed caryophyllideans in the basal clade of eucestodes, and supported the position of unsegmented tapeworms as the earliest divergent group. In the caryophyllidean clade, the family Lytocestidae was found to be polyphyletic group, with lingeages *Khawia* spp. and *Atractolytocestushuronensis* recovered as distantly related. *Atractolytocestushuronensis* clustered robustly with *Parabreviscolexniepini* and *Breviscolexorientalis* of the family Capingentidae. Thus, further study is needed to recircumscribe the Lytocestidae.

The mitogenome of *P.niepini* showed the consistent characters of unsegmented tapeworms determined by Li et al. (2017), and differed significantly from the segmented tapeworms in codon usage and gene order. The unsegmented caryophyllideans consisted of two clades according to the fish hosts, cypriniform and siluriform (Xi et al. 2018). The tapeworms sequenced in the present study were all collected from cypriniform fishes; however, specimens from catostomid fishes have never been reported. Further studies are required to determine the similarity of the mitogenome of caryophyllideans from catostomid fish.

## Conclusions

In this study, the complete mitogenome of the tapeworm *Parabreviscolexniepini* from a schizothoracine fish *Schizopygopsisyounghusbandi* was sequenced, annotated, and characterized. The mitogenome organization analysis indicated that it possessed a similar pattern to those caryophyllideans deposited in the GenBank database. Phylogenetic analysis based on mitogenomic data further confirmed the taxonomic validity of *P.niepini*, and its closest evolutionary relationship with *Breviscolexorientalis* and *Atractolytocestushuronensis*.

## Supplementary materials

Supplementary material 1 Table S1. Primers used to amplify and sequence the mitochondrial genomes of *Parabreviscolexniepini* Data type: molecular data This is an open access article distributed under the terms of the Creative Commons Attribution License (CC BY 4.0), which permits unrestricted use, distribution, and reproduction in any medium, provided the original author and source are credited.Bing-Wen Xi, Dong Zhang, Wen-Xiang Li, Bao-Juan Yang, Jun Xie

Supplementary material 2 Table S2. The list of cestodes species and outgroups used for comparative mitogenomic and phylogenetic analyses Data type: molecular data This is an open access article distributed under the terms of the Creative Commons Attribution License (CC BY 4.0), which permits unrestricted use, distribution, and reproduction in any medium, provided the original author and source are credited.Bing-Wen Xi, Dong Zhang, Wen-Xiang Li, Bao-Juan Yang, Jun Xie

Supplementary material 3 Table S3. Nucleotide composition and skewness of different elements of the studied mitochondrial genome Data type: molecular data This is an open access article distributed under the terms of the Creative Commons Attribution License (CC BY 4.0), which permits unrestricted use, distribution, and reproduction in any medium, provided the original author and source are credited.Bing-Wen Xi, Dong Zhang, Wen-Xiang Li, Bao-Juan Yang, Jun Xie

Supplementary material 4 Figure S1. Sequence alignment of trnS1 for *Parabreviscolexniepini* and other cestodes Data type: molecular data Explanation note: The position of the anticodon sequences was indicated. This is an open access article distributed under the terms of the Creative Commons Attribution License (CC BY 4.0), which permits unrestricted use, distribution, and reproduction in any medium, provided the original author and source are credited.Bing-Wen Xi, Dong Zhang, Wen-Xiang Li, Bao-Juan Yang, Jun Xie

Supplementary material 5 Figure S2. Relative Synonymous Codon Usage (RSCU) of *Parabreviscolexniepini* Data type: molecular data Explanation note: Codon families are labelled on the x-axis. Values on the top of the bars denote amino acid usage. This is an open access article distributed under the terms of the Creative Commons Attribution License (CC BY 4.0), which permits unrestricted use, distribution, and reproduction in any medium, provided the original author and source are credited.Bing-Wen Xi, Dong Zhang, Wen-Xiang Li, Bao-Juan Yang, Jun Xie

Supplementary material 6 Figure S3. Amino acid alignment for sites under positive selection for cytb Data type: molecular data Explanation note: The positions of each site are denoted on the top. An asterisk * denotes sites with posterior probability value ≥ 0.95 in Bayes Empirical Bayes (BEB) analysis. This is an open access article distributed under the terms of the Creative Commons Attribution License (CC BY 4.0), which permits unrestricted use, distribution, and reproduction in any medium, provided the original author and source are credited.Bing-Wen Xi, Dong Zhang, Wen-Xiang Li, Bao-Juan Yang, Jun Xie
