# Supplementary figures and images for: Characterization of the complete mitochondrial genome of Parabreviscolexniepini Xi et al., 2018 (Cestoda, Caryophyllidea)
Source: Zookeys. 2018 Sep 5;(783):97–112. doi: 10.3897/zookeys.783.24674 (PMC6182261; doi:10.3897/zookeys.783.24674)

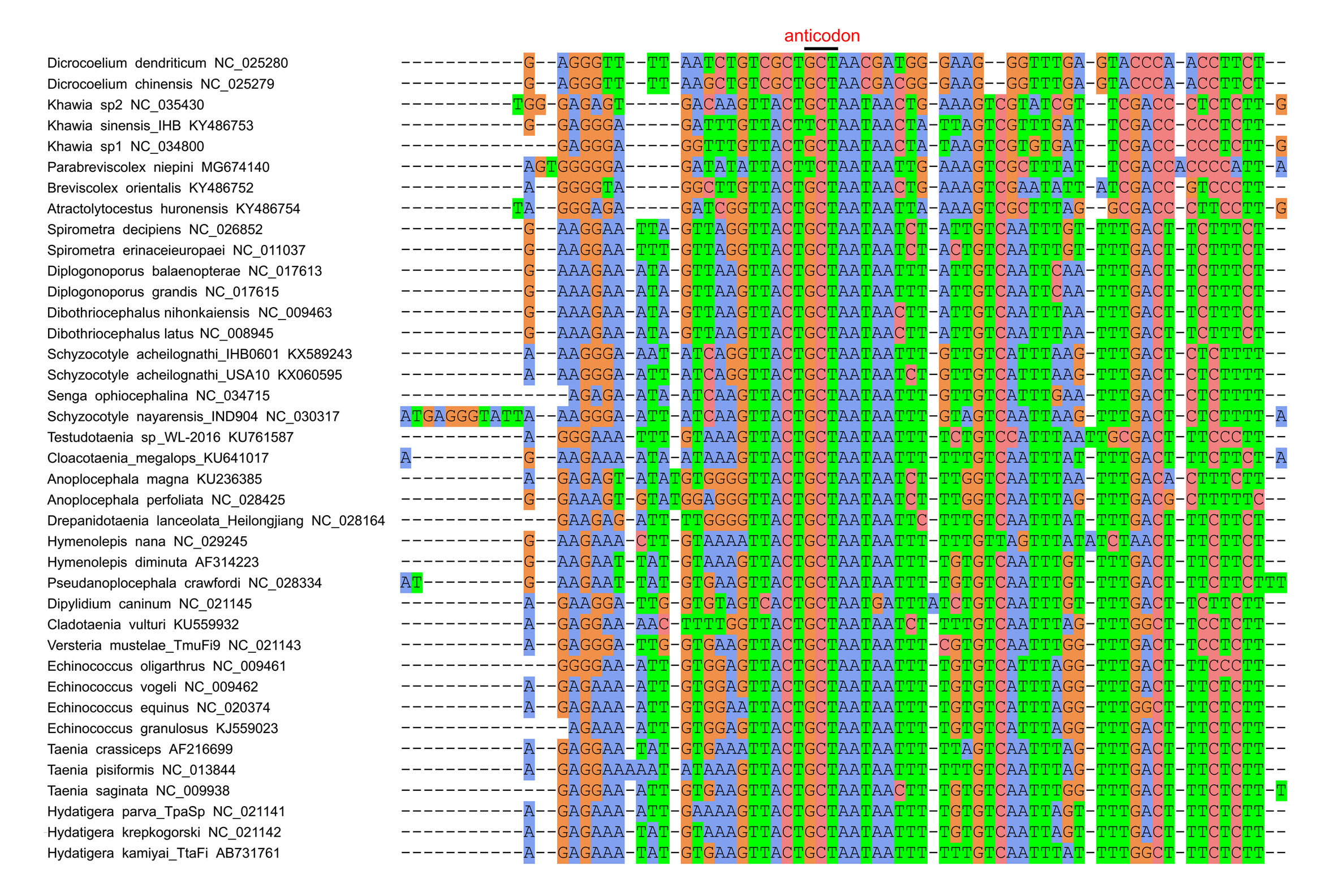

Supplement: Supplementary material 4 — Figure S1. Sequence alignment of trnS1 for Parabreviscolexniepini and other cestodes [file zookeys-783-097-s004.jpg]

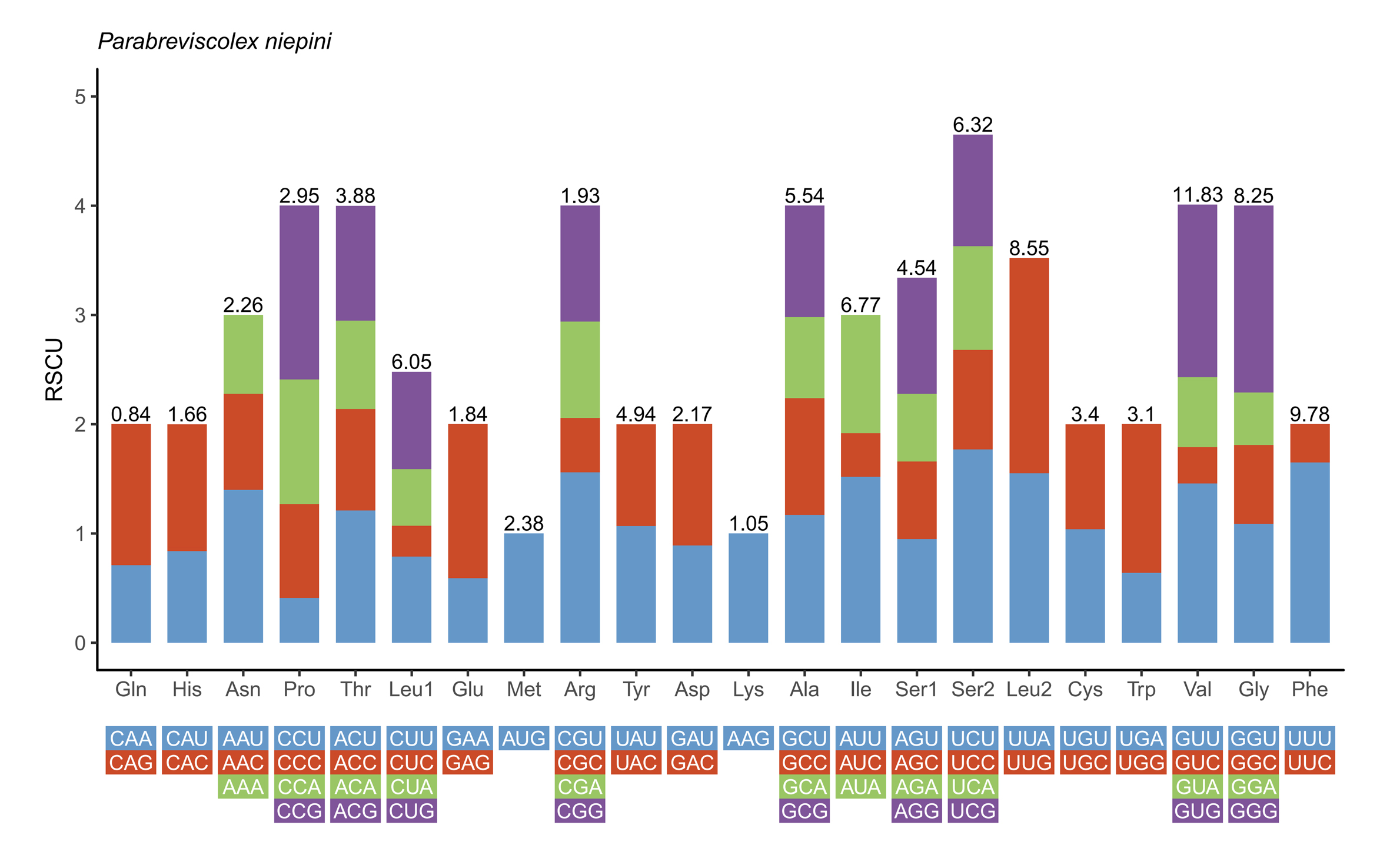

Supplement: Supplementary material 5 — Figure S2. Relative Synonymous Codon Usage (RSCU) of Parabreviscolexniepini [file zookeys-783-097-s005.jpg]

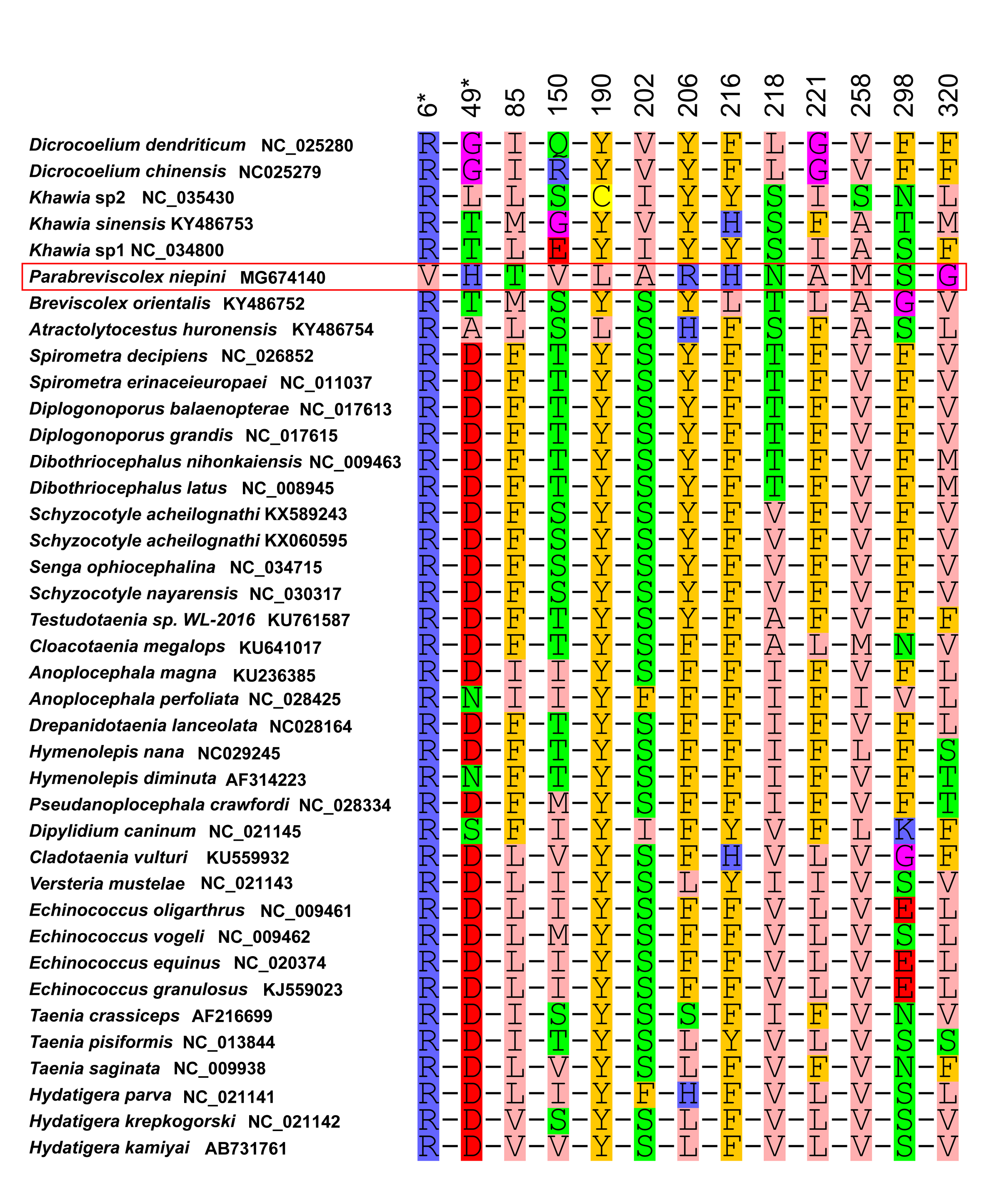

Supplement: Supplementary material 6 — Figure S3. Amino acid alignment for sites under positive selection for cytb [file zookeys-783-097-s006.jpg]
